# Cardiovascular and Renal Effects Induced by Alpha-Lipoic Acid Treatment in Two-Kidney-One-Clip Hypertensive Rats

**DOI:** 10.3390/biomedicines12081751

**Published:** 2024-08-03

**Authors:** Déborah Victória Gomes Nascimento, Darlyson Ferreira Alencar, Matheus Vinicius Barbosa da Silva, Danilo Galvão Rocha, Camila Ferreira Roncari, Roberta Jeane Bezerra Jorge, Renata de Sousa Alves, Richard Boarato David, Wylla Tatiana Ferreira e Silva, Lígia Cristina Monteiro Galindo, Thyago Moreira de Queiroz

**Affiliations:** 1 Laboratory of Nutrition, Physical Activity and Phenotypic Plasticity, Federal University of Pernambuco—UFPE, Vitória de Santo Antão 55608-680, Brazil; deborah.gnascimento@ufpe.br (D.V.G.N.); matheus.viniciusbarbosa@ufpe.br (M.V.B.d.S.); wylla.silva@ufpe.br (W.T.F.e.S.); ligia.mgalindo@ufpe.br (L.C.M.G.); 2 Department of Morphology, School of Medicine, Federal University of Ceará—UFC, Fortaleza 60430-160, Brazil; darlysonferreira_alencar@hotmail.com (D.F.A.); robertajeane@ufc.br (R.J.B.J.); renata.alves@ufc.br (R.d.S.A.); 3 Department of Physiology and Pharmacology, School of Medicine, Federal University of Ceará—UFC, Fortaleza 60430-160, Brazil; d.galvaorocha@gmail.com (D.G.R.); camilafroncari@ufc.br (C.F.R.); rdavid@ufc.br (R.B.D.)

**Keywords:** antioxidant, lipoic acid, hypertension, 2K1C, oxidative stress, renal function, renin–angiotensin system, vascular reactivity

## Abstract

α-Lipoic acid (LA) is an antioxidant of endogenous production, also obtained exogenously. Oxidative stress is closely associated with hypertension, which causes kidney injury and endothelial dysfunction. Here, we evaluated the cardiovascular and renal effects of LA in the two-kidney-one-clip (2K1C) hypertension model. The rats were divided into four groups: Sham surgery (Sham), the two-kidneys-one-clip (2K1C) group, and groups treated with LA for 14 days (Sham-LA and 2K1C-LA). No changes were observed in the pattern of food, water intake, and urinary volume. The left/right kidney weight LKw/RKw ratio was significantly higher in 2K1C animals. LA treatment did not reverse the increase in cardiac mass. In relation to vascular reactivity, there was an increase in the potency of phenylephrine (PHE) curve in the hypertensive animals treated with LA compared to the 2K1C group and also compared to the Sham group. Vasorelaxation induced by acetylcholine (Ach) and sodium nitroprusside (SNP) were not improved by treatment with LA. Urea and creatinine levels were not altered by the LA treatment. In conclusion, the morphological changes in the aorta and heart were not reversed; however, the treatment with LA mitigated the contraction increase induced by the 2K1C hypertension.

## 1. Introduction

Hypertension is the main risk factor for cardiovascular and kidney diseases and has been associated with a high risk of cardiovascular morbidity and mortality [1]. A decrease in blood pressure (BP) induces a reduction in cardiovascular risk [2]. Hypertension is a highly complex disease and several mechanisms underlie its pathophysiology, involving central nervous system imbalances, endothelial dysfunction, and renal defects [3].

Renovascular hypertension (RVH) has been widely studied as a prototype of angiotensin-dependent hypertension [4]. 2K1C is a classic animal model used in hypertension studies, as it significantly resembles renal hypertension as observed in humans [5]. The process of reducing renal flow induced by placing a clip on the renal artery promotes an increase in BP and vasoconstriction via the angiotensin-II (Ang-II) type 1 receptor (AT1R) [6].

Almost two decades ago, Griendling et al. (1994) [7] reported, for the first time, that Ang-II, a product of RAS cascade, activates vascular smooth muscle (VSM) nicotinamide adenine dinucleotide phosphate [NAD(P)H] oxidase, an important cellular source of reactive oxygen species (ROS), with a crucial role in inducing the state of oxidative stress [8].

It is possible to suggest that the use of experimental antioxidant therapy attenuates or prevents the development of hypertension through actions that include direct scavenging of ROS, mimicking antioxidants such as superoxide dismutase (SOD), and inhibiting the NAD(P)H oxidase enzyme [9,10,11,12,13], in addition to reducing the overexpression of AT1R [12,14] and attenuating cardiac and renal dysfunctions generated by hypertension [12,14].

Evidence has demonstrated that the use of antioxidants in the treatment of hypertension is promising. The therapeutic use of LA in hypertension is justified by its ability to restore the level of endogenous antioxidants and prevent the deleterious modification of the sulfhydryl group in Ca^2+^ channels [15]. Studies in experimental models of hypertension, including RVH, showed that treatment with LA was able to generate a variety of beneficial effects, involving reduced expression of NADPH oxidase subunits, in addition to attenuating sympathetic hyperexcitation [16], improving baroreflex sensitivity [10,11] and increasing SOD and glutathione (GSH) levels [17].

Furthermore, LA has been associated with an improvement in the endothelial synthesis of nitric oxide (NO), the leading vasodilator agent of the cardiovascular system [18,19,20], in addition to attenuating the increase in blood pressure by reducing the expression and activity of a disintegrin and metalloprotease 17 (ADAM17), an enzyme that promotes hypertensive effects through the release of a variety of inflammatory cytokines and is also responsible for the cleavage of angiotensin-converting enzyme type 2 (ACE2) [6,11].

Considering the biological importance of LA, the current study aimed to investigate the cardiovascular and renal effects induced by oral treatment with LA in 2K1C hypertensive rats.

## 2. Materials and Methods

### 2.1. Animals and Ethical Approval

Adult male Wistar rats (150–180 g) were housed in conditions of controlled temperature (21 ± 1 °C) and exposed to a 12 h light–dark cycle with free access to food (standard rodent pellets Nuvilab CR-1, Quimtia, Colombo, Brazil) and tap water. All procedures described in the present study are in accordance with the Institutional Animal Care and Use Committee of the Federal University of Ceará CEUA/UFC (protocol #2867020519). The animals were randomly allocated into four experimental groups, according to the surgical protocol performed and treatment: Sham surgery (Sham), n = 5; 2K1C (2K1C), n = 6; Sham surgery + lipoic acid (Sham-LA), n = 5; and 2K1C + lipoic acid (2K1C-LA), n = 5.

### 2.2. Induction of 2K1C Renovascular Hypertension

Rats underwent surgical procedures to develop renovascular hypertension (2K1C Goldblatt model), as described by Queiroz et al. (2012) [10]. Briefly, under combined ketamine and xylazine anesthesia (75 and 10 mg/kg, i.p., respectively), a midline abdominal incision was made. The left renal artery was exposed and isolated over a short segment by blunt dissection. A U-shaped silver clip (0.2 mm internal diameter) was placed over the vessel at a site proximal to the abdominal aorta and the wound closed and sutured. A Sham procedure, which entailed the entire surgery except for renal artery clipping, served as control. After the procedures, animals received an intramuscular injection of antibiotic (penicillin—24,000 IUs plus streptomycin—10 mg; Pentabiótico Veterinário—Zoetis, Campinas, São Paulo, Brazil) and an s.c. injection of analgesic/anti-inflammatory (ketoprofen 5 mg/kg; Agener União, Embu-Guaçu, Brazil). Rats were returned to their home cages and were observed for six weeks to develop hypertension.

### 2.3. Treatment with α-Lipoic Acid

LA was dissolved in a solution of NaOH (5N) and then with isotonic saline. A total of 28 days (4 weeks) after surgery to implant the clip in the renal artery (2K1C) or Sham surgery, the animals began treatment with oral administration, via gavage, of LA (60 mg/kg) or vehicle once a day for 14 days. During the second week (8th to 14th day) of treatment, animals were placed in metabolic cages. During this period, water, food intake and urine were measured daily.

### 2.4. Analysis of Water Intake, Food Intake, and Diuresis

From the 8th day of treatment, the animals were housed in metabolic cages; urine was collected through a container attached to the bottom of the cage, quantified using a graduated cylinder, and a sample was stored at −12 °C for biochemical analysis. Water and food intake were recorded daily. The daily food intake was measured from the difference between each final and initial chow weight remaining in the cage food container after a period of 24 h. The daily water intake was measured from the difference between each final and initial volume measured from a polypropylene bottle (100 mL capacity with divisions to the nearest mL) with a stainless-steel spout attached to the cage, after a period of 24 h.

### 2.5. Assessment of Blood Pressure

Blood pressure was assessed by the non-invasive method of tail plethysmography. Initially, the rats underwent an adaptation period of 3 days in a cylindrical container to minimize eventual bias in recordings due to stress. Initially, the rats were heated for 10 min in a heating box containing a 150-watt ceramic heat bulb to promote caudal artery dilation. Next, they were placed in the containment cylinder. An occluder and a sensor were fitted to the proximal portion of each rat’s tail. At the time of testing, they were coupled to an electric sphygmomanometer connected to a signal transduction system (MRBP System, IITC Life Science, Woodland Hills, CA, USA) and to a computer containing suitable software for continuous recording; then, the mean arterial pressure was calculated properly.

### 2.6. Histomorphometric Analyses

After removal, the kidneys (right and left) were sectioned in the sagittal plane; one-half was placed in histological cassettes for analysis and immersed in a container subjected to chemical fixation with 10% aqueous formalin solution, followed by dehydration with ethanol and soaking in paraffin. The paraffin blocks were submitted to microtomy with serial section, with a thickness of five micrometers. The other half was placed in centrifuge tubes for the study of oxidative stress.

The heart was sectioned transversely in the apex region, before being placed in a centrifuge tube for the same investigation as mentioned above, and the remainder was placed in Falcon tubes containing 10% formalin together with an abdominal aorta ring, sectioned transversely. The organs in the tubes were kept at −12 °C. Samples were stained with hematoxylin–eosin (HE).

The histological evaluation was carried out at the Center for Studies in Microscopy and Image Processing (NEMPI) of the Federal University of Ceará, and the slides were read by a single examiner.

Subsequently, the photomicrographs were analyzed using ImageJ software, version 1.44 (Research Services Branch, U.S. National Institutes of Health, Bethesda, MD, USA). All data were placed in an Excel spreadsheet for further statistical analysis. Images were captured using a light microscope coupled to a camera with an LAZ 3.5 acquisition system (Leica DM1000, Wetzlar, Germany).

#### 2.6.1. Aortic Lumen Analysis

To perform the analysis of the aortic lumen, ImageJ software was used. One (1) photomicrograph of each animal was taken, and the inner region was used for the study. This analysis is important because in hypertensive rats, the aortic lumen tends to be reduced in response to the increase in vascular resistance because of vasoconstriction.

#### 2.6.2. Measurement of the Tunica Adventitia/Media Ratio

The assessment of the proportion of collagen fiber deposition around the animals’ aortas was measured by the adventitial/mean ratio using HE staining. With the aid of an optical microscope coupled to the image acquisition system (LEICA), digital images were captured. According to the modified study carried out, the following measurements were taken: (A) outer area of the tunic adventitia, (B) outer area of the tunica media, and (C) inner area of the tunica media.

The difference between the outer area of the tunica adventitia and the outer area of the tunica media (A-B) results in the total area of the tunica adventitia. The difference between the outer and inner areas of the tunica media (B-C) results in the total area of the tunica media. From these results, the adventitial/mean ratio was calculated to analyze whether there was a change in the proportion of collagen fiber deposition around the artery.

In addition, we included another form of analysis in our study. The image of the aorta was divided into 4 (four) quadrants of equal size and dimensions. In each quadrant, we selected 2 (two) different regions to measure this ratio, thus making 8 microphotographs in each aortic ring.

#### 2.6.3. Area and Volume of Cardiomyocytes

To assess the areas and volumes of cardiomyocytes, 5 (five) photomicrographs of each heart were taken. To obtain the morphometric data from the images, longitudinal sections of muscle bundles of the heart were performed. Samples were stained in HE.

Subsequently, for volume analysis, with the aid of a mouse and ImageJ software, the smallest and largest diameters of 5 cardiomyocytes per image were measured to assess cell activity, and the values were applied in the formula v = a2. b/1.91, where a = smallest diameter, b = largest diameter, and 1.91 is a constant. To assess the area, using ImageJ software and with the aid of a mouse, the outer edge of the cardiomyocytes was outlined, thus making up the entire area.

### 2.7. Biochemical Analysis of Urine

Bioclin (Química Básica LTDA., Belo Horizonte, MG, Brazil) commercial kits were used for the dosages of biochemical markers. The measurement of plasma and urinary creatinine was performed using the modified Jaffé method and, for urea, a kinetic UV urea kit. The tests were carried out with BS 120 automated equipment using spectrophotometry.

### 2.8. Vascular Reactivity Study

Following evaluation of the functional vascular endothelium, two contractions were induced using depolarizing Tyrode’s solution (K^+^ 60 mmol·L^−1^). After washing out the responses to high K^+^, cumulatively increasing concentrations of PHE (0.1 nmol·L^−1^–10 mmol·L^−1^) were added to the aortic rings with and without endothelium to evaluate the contractile responses. To evaluate the relaxation responses, acetylcholine (ACh, 0.1 nmol·L^−1^–10 µmol·L^−1^) in rings with endothelium or sodium nitroprusside (SNP, 0.1 pmol·L^−1^–1 µmol·L^−1^) in rings without endothelium were added to the bath. Concentration-dependent contractile responses to PHE were recorded as a percentage of the maximum contraction obtained following tissue stimulation with high K^+^. Relaxation responses to cumulative concentrations of ACh and SNP were calculated as a percentage of inhibition of the PHE-induced maximal contraction. In vascular reactivity experiments, we use the parameters of efficacy (MR—the maximum response, expressed in %) and potency (pEC50 = the negative logarithm to base 10 of the EC50 of an agonist, which is the molar concentration of an agonist that produces 50% of the maximal possible effect of that agonist). Relaxation responses to cumulative concentrations of ACh and SNP were calculated as a percentage of inhibition of the PHE-induced maximal contraction. In vascular smooth muscle relaxation tests, the relaxing effect (R) of the substances was calculated, for each concentration, as a function of the maximum contraction provided by the agonist, according to the expression R = ((T_A_ – T_S_)/T_A_) × 100, where T_A_ and T_S_ are, respectively, the tensions resulting from the action of the agonist (PHE) and a given substance (ACh or SNP). The graphs were then created based on the average values of the magnitude of the vasodilator or vasoconstrictor effect, calculated for each concentration of the substance (after logarithmic transformation). Such data were used to construct concentration–effect curves using nonlinear regression analysis. To address this, the model that uses a sigmoid function of the type was taken as a basis, y = a + (b − a)/(1 + 10^((logCE50−x)·s)^), where y corresponds to the response measure (relaxing effect), x to the decimal logarithm of the concentration, a to the minimum response, and b to the maximum response.

### 2.9. Statistical Analysis

Values are expressed as mean ± SEM. Statistical analysis was performed using one-way analysis of variance ANOVA followed by Tukey’s multiple comparison post hoc test. We used the Shapiro–Wilk test to verify the normality of data. The statistical analyses and graphs were performed and constructed using GraphPad Prism version 8.0 (GraphPad Software Corporation San Diego, CA, USA). The differences between groups were considered significant at *p* < 0.05.

## 3. Results

### 3.1. Effect of α-Lipoic Acid Treatment on Water Intake and Urine Levels in 2K1C Rats

**Figure 1:**
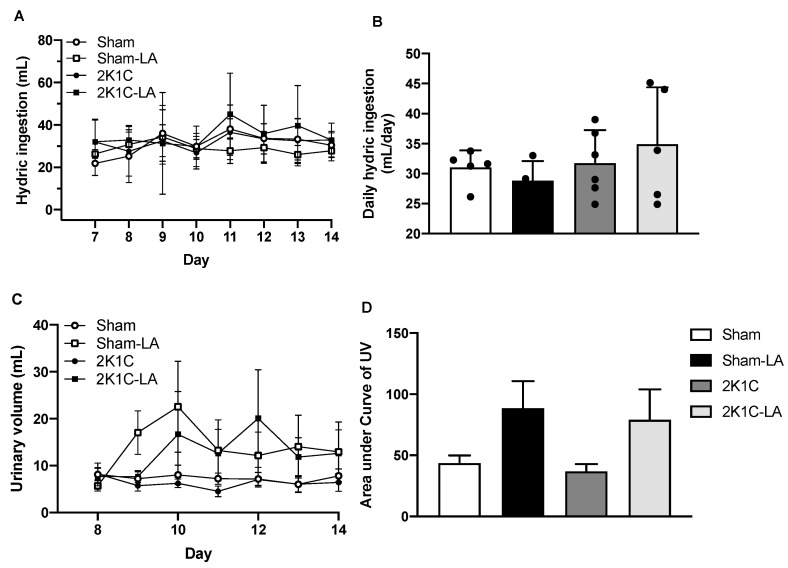
Effects of LA treatment on water intake and urinary levels. (**A**) Daily water intake, (**B**) mean water intake, (**C**) temporal progression of urinary volume, and (**D**) urine volume. Data are expressed as mean ± SEM. Comparisons between groups by one-way ANOVA associated with Tukey’s post-test.

The daily water intake (Figure 1A) did not change among the experimental groups, which presented the following mean values after 14 days of treatment with LA: Sham (31.00 ± 2.89 mL/24 h), Sham-LA (28.84 ± 3.25 mL/24 h), 2K1C (31.73 ± 5.50 mL/24 h), and 2K1C-LA (34.88 ± 9.48 mL/24 h) (Figure 1B).

The daily urinary volume was analyzed (Figure 1C) counting from the 1st day that the animals remained in the metabolic cage, that is, the beginning of the 2nd week of treatment. No significant changes in the urinary volume were observed between groups (Figure 1D).

### 3.2. Effect of α-Lipoic Acid Treatment on Food Intake in 2K1C Rats

**Figure 2:**
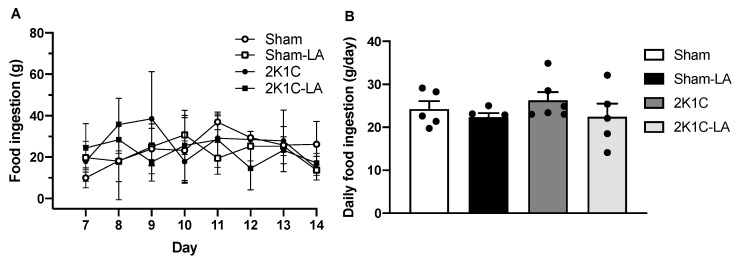
Effects of LA treatment on food intake. (**A**) Temporal progression of food intake and (**B**) average daily food intake. Data are expressed as mean ± SEM. Comparisons between groups by one-way ANOVA associated with Tukey’s post-test.

Daily food intake (Figure 2A) was also measured from the 1st day the animals remained in the metabolic cage, in parallel with the start of the 2nd week of treatment. No differences were observed between the analyzed groups, although the 2K1C group presented an ingestion peak on the 2nd (35.67 ± 12.72 g) and 3rd days (38.50 ± 22.76 g), before normalizing the next day.

The average daily food intake presented the following values: Sham (24.23 ± 4.21 g/24 h), Sham-LA (22.16 ± 2.46 g/24 h), 2K1C (26.27 ± 4.71 g/24 h), and 2K1C-LA (22.45 ± 6.84 g/24 h). There was a tendency of the treated animals to ingest less food during the study; however, these values did not show a significant difference (Figure 2B).

### 3.3. Blood Pressure Measurement and Relationship between Kidney/Body Weight and Renal Index

**Figure 3:**
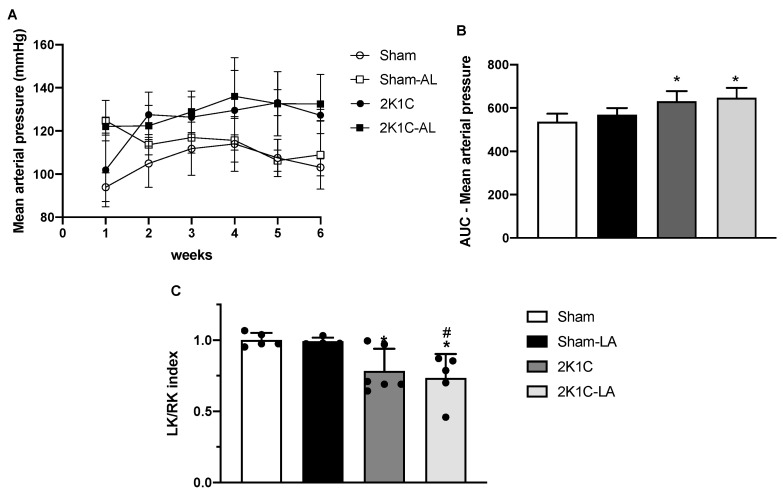
Effects of LA treatment on mean arterial pressure during the weeks (**A**) and the area under the curve graph of the MAP (**B**). Graph (**C**) represents the left kidney (LK)/right kidney (RK) ratio. Data expressed as mean ± SEM. Comparisons between groups by one-way ANOVA associated with Tukey’s post-test. * Denotes a significant difference concerning the Sham group (*p* < 0.05). # Denotes a significant difference in relation to the Sham-LA group (*p* < 0.05).

The successful induction of 2K1C surgery was confirmed by the blood pressure analysis using the non-invasive method of tail plethysmography. We can note that there was a greater mean arterial pressure (MAP) through the six weeks of observation in the 2K1C compared to the Sham group as observed in the area under curves of MAP (631.1 ± 19.0 vs. 536.6 ± 16.4, respectively, *p* > 0.05). The hypertensive animals treated with LA did not reverse the increase in MAP (647.2 ± 20.4 vs. 631.1 ± 19.0, respectively) (Figure 3A,B).

In order to confirm whether the reduction in blood flow in the renal artery of the clipped kidney could be a parameter to assess RVH, the weights of the kidneys in relation to the body weight of the animals and the LKw/RKw ratio were calculated (Figure 3).

After weighing the kidneys, it was found that the Sham (1.00 ± 0.05) and Sham-LA groups (0.99 ± 0.03) presented kidney weights in the proportion of 1:1, demonstrating that there was no significant change between them. Between the 2K1C (0.78 ± 0.16) and 2K1C-LA (0.73 ± 0.17) groups, there was also no significant change.

However, there was a reduction in the renal index in both the 2K1C and 2K1C-LA groups compared to the Sham group, demonstrating that hypertensive animals in the 2K1C hypertension model presented a reduction in this index in relation to normotensive animals.

**Figure 4:**
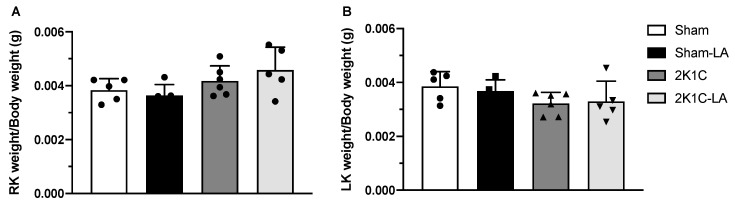
Effects of LA treatment on relationship between kidney weight and body weight. (**A**) Right kidney/body weight ratio and (**B**) left kidney/body weight ratio. Data expressed as mean ± SEM. Comparisons between groups by one-way ANOVA associated with Tukey’s post-test.

Each kidney in its referred group was analyzed separately. We found that the right kidneys of the 2K1C (0.0042 ± 0.0006 g) and 2K1C-LA (0.0046 ± 0.0009 g) groups, when compared to the Sham (0.0038 ± 0.0004 g) and Sham-LA groups (0.0037 ± 0.0004 g), showed a tendency to exhibit compensatory hypertrophy, but this was not significant (Figure 4A).

The left kidneys of the 2K1C (0.0032 ± 0.0004 g) and 2K1C-LA (0.0033 ± 0.0008 g) groups compared to the Sham (0.0039 ± 0.0005 g) and Sham-LA (0.0037 ± 0.0004) groups showed a tendency to exhibit hypotrophy, which is explained by the RVH model used in this study, which promotes a reduction in renal blood flow without causing ischemia. However, neither these results nor those of the right kidneys were significant (Figure 4B).

### 3.4. Effect of α-Lipoic Acid Treatment on Weight and Cardiac Morphology in 2K1C Rats

**Figure 5:**
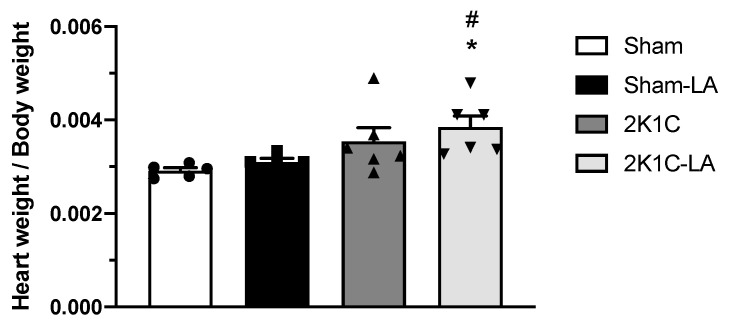
Effects of LA treatment on relationship between heart weight and body weight. Data expressed as mean ± standard deviation. * Denotes a significant difference with the Sham group; # vs. Sham-LA group (*p* < 0.05). Data expressed as mean ± SEM. Comparisons between groups by one-way ANOVA associated with Tukey’s post-test.

No significant changes were observed in the relationship between heart and body weight from the animals in the 2K1C (0.0035 ± 0.0007 mg/g) and Sham (0.0029 ± 0.0001 mg/g) groups. However, animals in the 2K1C-LA group (0.0039 ± 0.0006 mg/g) compared to the Sham (0.0029 ± 0.0001 mg/g) and Sham-LA groups showed a significant increase (*p* < 0.05) in the relationship between heart/body weight, which indicates the presence of a hypertrophic process in the organ after renovascular surgery and the absence of reversal with LA treatment (Figure 5).

### 3.5. Treatment with α-Lipoic Acid Promotes Changes in Vascular Reactivity in 2K1C Rats

**Figure 6:**
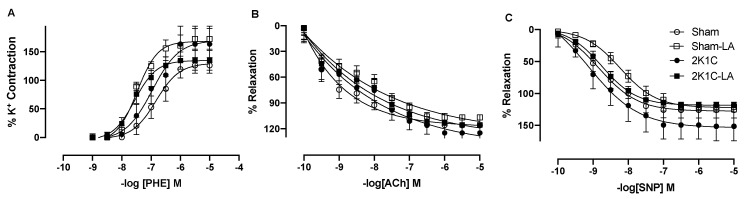
Effects of alpha lipoic acid treatment on endothelium-dependent and -independent relaxation and contractile response to phenylephrine in 2K1C rats. Concentration–response curves for increasing concentrations of (**A**) acetylcholine (ACh), (**B**) phenylephrine (PHE), and (**C**) sodium nitroprusside (SNP). Values are expressed as mean ± SEM. Comparisons between groups by one-way ANOVA associated with Tukey’s post-test.

In relation to vascular reactivity, no significant difference was observed in PHE curve related to maximum response (MR) (Figure 6A and Table 1). However, there was an increase in the potency (pD2) of PHE curve in the hypertensive animals treated with LA compared to the 2K1C group (7.52 ± 0.09 vs. 6.97 ± 0.15, respectively, *p* > 0.05, n = 6), and also compared to the Sham group (7.52 ± 0.09 vs. 6.84 ± 0.10, respectively, *p* > 0.05, n = 6) (Figure 6A and Table 1).

In vitro pharmacological tests were performed on isolated aortic rings with intact endothelium (ACh). Evaluation of pD2 values among the respective groups: Sham (9.05 ± 0.09), Sham-LA (8.58 ± 0.09), 2K1C (8.64 ± 0.14), and 2K1C-LA (8.64 ± 0.13). When evaluating MR (116.0 ± 2.1%, 106.8 ± 2.5%, 124.9 ± 8.9% and 115.4 ± 11.6%, respectively, n = 6), the results demonstrate that the LA treatment was not able to significantly improve vasorelaxation for the ACh in hypertensive animals (Figure 6B and Table 1).

In the aortic rings without endothelium, in the vasorelaxation curve for the SNP, no significant difference was observed when we evaluated pD2 (8.70 ± 0.10), Sham-LA (8.19 ± 0.06), 2K1C (8.74 ± 0.17), and 2K1C-LA groups (8.65 ± 0.04) or when evaluating MR in the respective groups (126.0 ± 12.3%, 121.0 ± 3.9%, 151.5 ± 22.3%, 118.1 ± 2.5%, n = 6) (Figure 6C and Table 1).

### 3.6. Effect of Alpha Lipoic Acid Treatment on Urinary Biochemistry in 2K1C Rats

**Figure 7:**
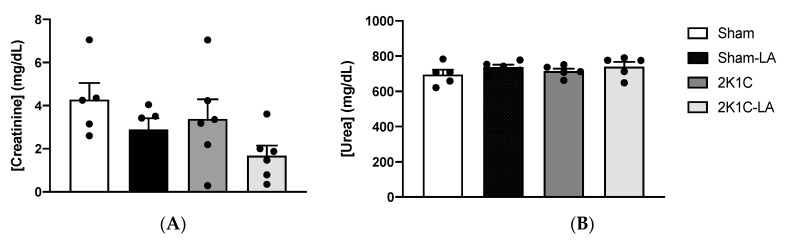
Analysis of renal function according to urinary creatinine and urea clearance. Creatinine concentration (**A**) and urea concentration (**B**). Comparisons between groups by one-way ANOVA associated with Tukey’s post-test (2K1C-LA vs. 2K1C group, *p* = 0.3116; 2K1C-LA vs. Sham group, *p* = 0.0771).

There was no significant difference in creatinine levels between groups: Sham (4.29 ± 1.98 mg/Dl), Sham-LA (2.77 ± 1.30 mg/Dl), 2K1C (4.45 ± 1.79 mg/Dl), and 2K1C-LA (1.69 ± 1.46 mg/Dl) (Figure 7A). There were also no significant differences in urea levels: Sham (695.80 ± 61.24 mg/Dl), Sham-LA (742.30 ± 35.33 mg/Dl), 2K1C (714.40 ± 33.64 mg/Dl), and 2K1C-LA (740.60 ± 59.22 mg/Dl) (Figure 7B). This finding suggests that treatment with LA did not promote effects on renal function.

### 3.7. Effect of Alpha α-Acid Treatment on Aortic and Cardiac Morphology in 2K1C Rats

**Figure 8:**
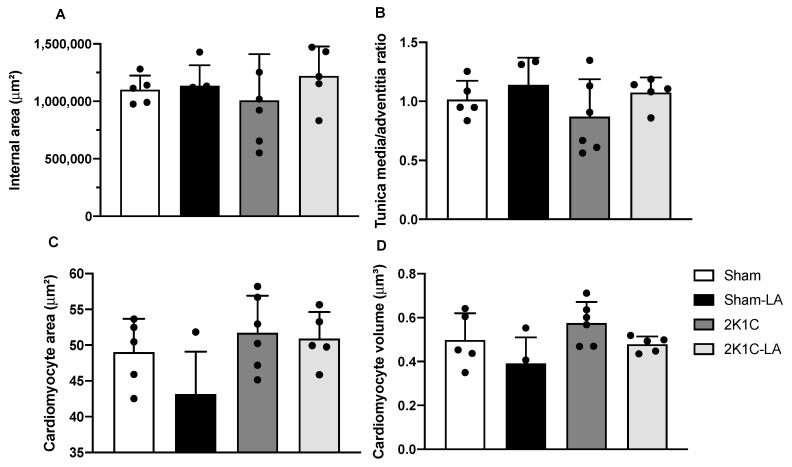
Histomorphometric analysis of the heart and aorta. (**A**) Aortic lumen area, (**B**) tunica adventitia/media ratio, (**C**) cardiomyocyte area, (**D**) cardiomyocyte volume. Comparisons between groups by one-way ANOVA associated with Tukey’s post-test.

To evaluate the effects of LA treatment on cardiac and aortic morphology, photomicrographs were captured and analyses were performed using ImageJ software. Although we did not find significant changes in any of the studied groups, the 2K1C group (1,008,276.00 ± 402,505.00 µm) and the 2K1C-LA group (1,220,547.00 ± 256,010.00 µm) did not present changes as many changes in the aortic lumen compared to the Sham group (1,100,162.00 ± 124,539.00 µm) (Figure 8A).

**Figure 9:**
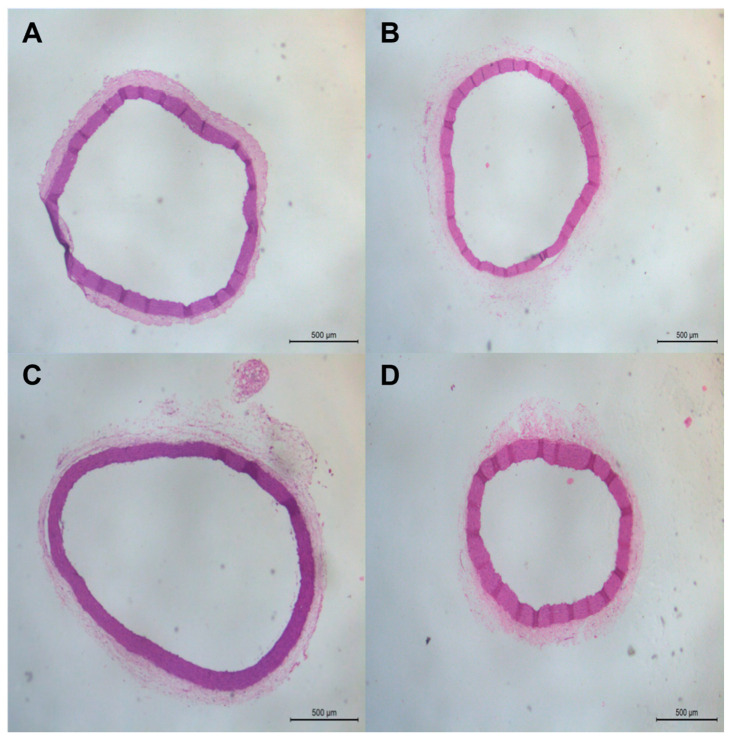
Histological analysis of abdominal aorta ring determined by HE. (**A**) Sham, (**B**) Sham-LA, (**C**) 2K1C, and (**D**) 2K1C-LA. Scale bar  =  500 μm, 100×.

The evaluation of the proportion of collagen fiber deposition around the animals’ aortas was measured by the adventitia/mean ratio through HE staining. The 2K1C group (1.39 ± 0.48 µm) and the 2K1C-LA group (1.28 ± 0.31 µm) did not show alterations in collagen deposition compared to the Sham group (1.09 µm ± 0.13) (Figure 8B and Figure 9).

**Figure 10:**
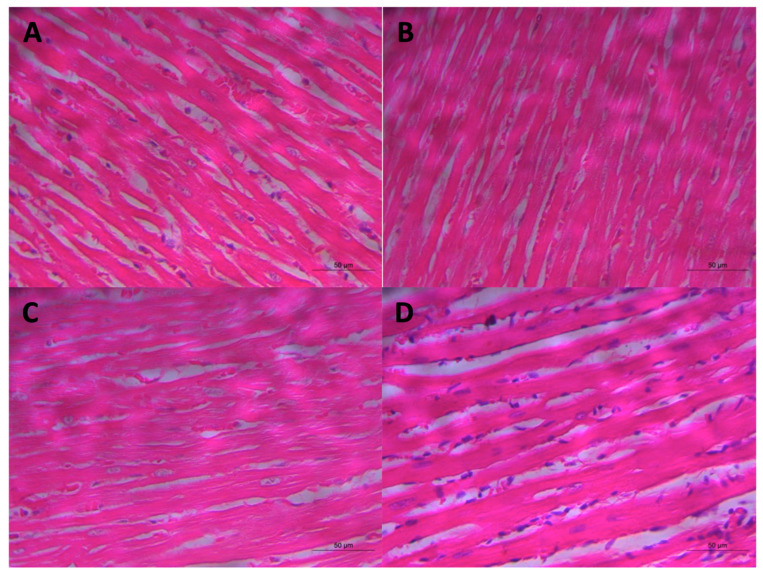
Histological analysis of heart determined by HE. (**A**) Sham, (**B**) Sham-LA, (**C**) 2K1C, and (**D**) 2K1C-LA. Scale bar =  50 μm, 400×.

We did not find significant differences in cardiomyocyte areas between the groups: Sham (49.01 ± 4.68 µm), Sham-LA (50.90 ± 3.74 µm), 2R1C (51.75 ± 5.18 µm), and 2K1C-LA (50.90 ± 3.74 µm) (Figure 8C). Likewise, for cardiomyocyte volume results, Sham (0.50 ± 0.12 µm), Sham-LA (0.39 ± 0.12 µm), 2K1C (0.58 µm ± 0.10), and 2K1C-LA (0.48 ± 0.04 µm) (Figure 8D). While no significant differences were identified, the increased area and volume values in the hypertensive groups infer the presence of cardiac hypertrophy in these animals, unlike normotensive animals, demonstrating that the treatment was not able to reduce the area and volume of cardiomyocytes (Figure 10).

## 4. Discussion

Our results demonstrated that oral treatment with LA induced an increase in the potency of PHE curve in the hypertensive animals treated with LA compared to the 2K1C group and also compared to Sham group. However, it was observed that the treatment did not promote a significant change in the concentration–response curve for ACh and SNP, indicating that LA did not restore the vasorelaxation altered by 2K1C hypertension.

The findings in the present research corroborate the study by Queiroz et al. (2012) [10], which showed that 2K1C animals treated with LA at the same dose presented a reduction in blood pressure compared to untreated 2K1C. This decrease in BP occurred due to the improvement in baroreflex sensitivity in 2K1C animals treated with the antioxidant [10].

An important finding of this study was that treatment with LA increased the contractile response in animals submitted to Sham surgery. This fact, as far as we could establish, has not yet been described in the literature. Contrary to the results for vasorelaxation obtained in this study, another study carried out with a different experimental model in diabetic rats using the streptozotocin injection method and fed with a high-fat diet, the treatment with LA was able to improve vascular reactivity in aortic rings, increasing ACh vasorelaxation, and improving vascular function through pathways that increase hydrogen sulfide, a gaseous transmitter with a beneficial effect on the vascular system, in addition to decreasing vascular smooth muscle cell autophagy through regulation of the AMPK/mTOR pathway [21].

Concerning the investigation of the renal effects of the treatment, after analyzing the ratio between the weights of the kidneys, it was verified that the 2K1C animals had a lower LKw/RKw ratio than the Sham animals. This result corroborates another study in which this relationship was evaluated in rats after 6–8 weeks, similar to our renal clipping protocol. The authors demonstrated that animals with an intermediate ratio, between the range of 0.5 to 0.8, were 100% hypertensive, with blood pressure greater than 150 mmHg, whereas for animals with a ratio lower than 0.4 and greater than 0.9, less than 50% were hypertensive [22]. Therefore, the LKw/RKw ratio found in 2K1C rats in the present study suggested changes in renal morphology after the development of RVH, as shown in other studies [23,24,25].

Regarding the qualitative and quantitative assessment of the excretion capacity of the kidney, the glomerular filtration rate is the most commonly used measure for such purposes, being more faithfully obtained during clinical practice from the creatinine clearance, and is performed in urine collected in precisely 24 h [25]. This is one of the most widely used methods in preclinical studies to assess the function and presence of renal dysfunction. It is performed by estimating the glomerular filtration rate, considering plasma or serum levels of urea and creatinine [26,27]. However, in the current study, we used 24 h urine for the individual evaluations. Studies have demonstrated that LA decreased the renal tubular injury scores and urinary damage markers and increased glomerular filtration [20]. The augment in glomerular filtration leads to an increase in urinary volume, which can be an explanation of the UV data presented in our study.

Chronic creatinine levels in animals treated with LA did not show a significant difference compared to untreated animals. Studies evaluating the renoprotective and cardioprotective effects of treatment with LA observed a decrease in serum creatinine levels [28]. In addition, another biomarker of kidney injury was evaluated, urea level, and showed that our treatment also did not affect the reduction in urea between the groups, demonstrating that in these animals there was no commitment of renal function. Contrary to what was observed in the study by Amat et al. (2014) [29], 2K1C hypertension promoted renal dysfunction in animals, evidenced by increased serum levels of creatinine and urea. Although we did not measure the serum values of these markers, we used the values as a basis since urea and creatinine values reported through 24 h urine analyses are scarce in the literature.

Classically, the increase in serum creatinine, oliguria, albuminuria, and electrolyte abnormalities are considered indicators of kidney injury, due to tubular changes or even structural damage that can only be visualized by imaging or histology exams [19]. However, in the kidney injury model caused by cisplatin [18], the authors reported an increase in glomerular filtration, with increased creatinine clearance and consequent reduction in plasma creatinine, in addition to the attenuation of oxidative damage. Therefore, even though there is a tendency for creatinine to decrease, it is not possible to affirm, in the studied model, that there was significant renal protection with the use of lipoic acid. Likewise, no histological changes were observed that support this finding

The 2K1C model is described as a model of hypertension independent of the increase in volume and vasopressin secretion, since the remaining non-clipped kidney acts in a compensatory way [30]. Our behavioral results involving daily food and water intake and daily diuresis did not show significant differences between groups. These findings are contrary to what was found in other studies [31,32], in which an increase in water intake and diuresis was demonstrated in 2K1C animals when compared to control animals. The divergence in behavioral patterns observed among those studies and in the present results might be due to the different rat strain used in the experiments.

Heart diseases have a common characteristic, accompanied mainly by increased myocardial mass. This hypertrophy occurs through the absolute or relative thickening of the walls of the chambers due to the increase in the dimensions of the cardiomyocytes, especially pathologically in conditions of prolonged and abnormal hemodynamic stress, such as hypertension and myocardial infarction [33,34].

It is understood that cardiac hypertrophy is expected in this experimental model of hypertension. However, this increase was not seen in the 2K1C animals. In a study using the same model, renal stenosis caused cardiac hypertrophy, accompanied by increased collagen deposition and cardiomyocyte diameter [35]. Therefore, we also decided to conduct a cardiac evaluation using the dimensions of the cardiomyocytes and concluded that the area and volume of the cardiomyocytes did not present significant alterations among the studied groups.

Similarly, in a study carried out with SHR, the 30-day treatment with the enantiomer -(−)-ALA (125 µmol/kg/day) did not reverse cardiac hypertrophy and fibrosis generated by hypertension. However, in animals treated with another enantiomer, (+)-ALA (125 µmol/kg/day), the attenuation of left ventricular fibrosis was observed [36].

The aortic wall consists of three concentric layers: the tunica intima, tunica media, and tunica adventitia [37]. The biomechanical properties of vessels, large arteries, and veins largely depend on the amount and balance between extracellular matrix constituents, such as collagen and elastin, and local proteases, such as matrix metalloproteinases and leukocyte elastase. This balance can be impaired in the presence of vascular pathologies, such as hypertension [38,39].

From this, it was decided to evaluate the adventitia/mean ratio to evaluate the collagen deposition around the aortas of the Sham and 2K1C groups. We observed that the 2K1C rats tended to accumulate more collagen fibers, whereas the treatment with LA showed a possible tendency to reduce this deposition. However, the results were not significant. Similarly, a study carried out with the descending thoracic aorta of a rabbit model with aortic valve calcification demonstrated that treatment with LA protected against medial vascular calcification but did not prevent the increase in arterial wall thickness [40].

Understanding the histological alterations caused by hypertension, we sought to analyze the area/inner lumen of the aorta in our animals. Our results showed a reduction in this lumen in 2K1C animals, while in the presence of LA, this lumen returned to its normal morphology. This alteration may be explained by smooth muscle cells, which can rearrange themselves around a reduced arterial lumen, accompanied by a more significant deposition of extracellular matrix, which can lead to a decrease in biomechanical and hemodynamic function, consequently compromising tissue perfusion [39,41], which was supposedly verified in the adventitious/mean ratio.

## 5. Conclusions

Our results indicate that the treatment promoted little effects on the cardiovascular system, showing that LA was not able to decrease the augment of mean arterial pressure induced by renovascular hypertension. LA also increased the vasoconstriction evoked by PHE in animals with renovascular hypertension compared to the 2K1C and Sham groups. Although we observed changes in urinary volume and creatinine levels induced by LA, these responses are not significant; this was the same for the morphology alterations. In this context, further investigation is needed to examine the detailed mechanisms related to the cardiovascular and renal actions of LA.

## Figures and Tables

**Table 1:** Table showing the MR expressed in % as well as the pD2 values from different treatments in normotensive and hypertensive rats using PHE, Ach, and SNP.

(A) MR (%)	Sham	Sham-LA	2K1C	2K1C-LA
PHE	126.2 ± 13.9	172.3 ± 18.0	163.2 ± 11.6	134.7 ± 17.2
ACh	116.0 ± 2.1	106.8 ± 2.5	124.9 ± 8.9	115.4 ± 11.6
SNP	126.0 ± 12.3	121.0 ± 3.9	151.5 ± 22.3	118.1 ± 2.5
**(B) pD2**	**Sham**	**Sham-LA**	**2K1C**	**2K1C-LA**
PHE	6.84 ± 0.10	7.40 ± 0.07	6.97 ± 0.15	7.52 ± 0.09 *^,&^
ACh	9.05 ± 0.09	8.58 ± 0.09	8.64 ± 0.14	8.64 ± 0.13
SNP	8.70 ± 0.10	8.19 ± 0.06	8.74 ± 0.17	8.65 ± 0.04

* Denotes a significant difference in relation to the Sham group. ^&^ Denotes a significant difference in relation to the 2K1C group.

## Data Availability

Data are contained within the article.
